# The impact of socioeconomic status on the prevalence of antimicrobial resistance in high-income nations: a systematic review

**DOI:** 10.1017/ash.2025.10177

**Published:** 2025-10-14

**Authors:** Ethan Levitch, Levi Matthews, Eugene Choi, Sagaana Thushiyenthan, Lisa Hall, Jake Tickner, Amalie Dyda

**Affiliations:** 1 University of Queensland Medical School, Brisbane, QLD, Australia; 2 The School of Public Health, University of Queensland, Brisbane, QLD, Australia; 3 UQ Centre for Clinical Research, University of Queensland, Brisbane, QLD, Australia

## Abstract

**Objective:**

Antimicrobial resistance (AMR) poses an escalating global threat, transforming once-treatable infections into major health challenges. Although antibiotic misuse is a well-known driver of AMR, particularly in low- and middle-income settings, the silent epidemic may be fueled by socioeconomic disparities even in high-income countries. This systematic review investigates the relationship between socioeconomic status (SES) and AMR prevalence across high-income nations based on the World Bank classification.

**Design:**

The studies included in this review span multiple observational designs (cross-sectional, cohort, and case-control) across various high-income nations, assessing the association between SES indicators (eg, income, education, and household crowding) and AMR strains, primarily methicillin-resistant *Staphylococcus aureus* (MRSA) and multidrug-resistant *Escherichia coli*.

**Results:**

Findings consistently indicate that lower SES correlates with higher AMR prevalence, particularly in MRSA infections (*r* = 0.76, Blakiston et al). The review highlights that indices of SES (often derived from government census data) are consistently associated with lower income, lower educational attainment, and increased household density with elevated AMR prevalence.

**Conclusion:**

The variability among studies in SES metrics, including income measures and deprivation indices, limits generalizability. Exceptions to this trend, noted in select studies focusing on distinct AMR strains like ceftriaxone-resistant *E. coli*, underscore the complexity of SES-related AMR mechanisms. This review supports public health initiatives aimed at targeting low-SES communities with AMR mitigation strategies, advocating for continued monitoring and intervention to curb AMR spread in vulnerable populations.

## Background

Antimicrobial resistance (AMR) arises when microorganisms adapt to survive antimicrobial drugs through spontaneous genetic mutations,^1,2^ a process exacerbated by human activities such as the misuse of antimicrobials and poor infection control practices.^3,4^ In 2019, approximately 4.95 million deaths occurred globally in direct association with AMR.^5^ In Australia, AMR-associated deaths have risen to over 1,000 annually, and the 5 most common AMR pathogens were attributed to the loss of 27,705 quality-adjusted life years in 2020.^6^ The most prominent of these were methicillin-resistant *Staphylococcus aureus* (MRSA) infections in the respiratory tract, bloodstream, and skin, accounting for $3.5 billion of Australian healthcare expenditure.^7^ Globally, AMR threatens the efficacy of life-saving antibiotics, fueling community-wide colonization with resistant pathogens that were once confined to hospital settings.^8^

At this time, there is sparse literature that evaluates low socioeconomic status (SES) as a key driver of AMR, limiting its recognition as a potential target for policy and intervention. Numerous primary studies have examined the prevalence of resistant strains of specific pathogens in clinical settings, although current literature lacks consensus on SES as an independent contributor to AMR in high-income countries. SES is a composite of contributing determinants, and the term SES represents the factors collectively (household crowding, education level, etc) unless otherwise specified.

At the community level, low-SES neighborhoods may be disproportionately vulnerable to AMR due to reduced gut microbiome diversity linked to food insecurity.^9^ Determinants of SES such as household crowding (per Canadian National Occupancy Standard) increase exposure to microorganisms and facilitate resistance development. Education level directly correlates with health literacy, influencing antimicrobial use and treatment adherence.^10^ Furthermore, lower SES communities have a greater reliance on Medicare and its international counterparts, which stresses stewardship and delays treatment in the community.^11^

In rural and remote areas, lack of stringent antimicrobial stewardship (AMS) contributes significantly to inappropriate prescription and antibiotic use,^12^ directly increasing AMR. Additionally, crowded housing, inadequate hygiene practices, limited healthcare access, and literacy add to increased AMR in rural communities.^6^ Less studied, however, are populations of low SES in urban and suburban communities with similar AMR prevalence and related disease burdens. Local studies show direct correlations between low SES and the prevalence of specific strains of AMR pathogens, suggesting that similar social determinants drive these disparities in both rural and urban areas.^12,13^

Examining SES in specifically high-income countries is a crucial distinction when identifying global patterns in AMR. Low- and middle-income countries (LMICs) have severe limitations to antimicrobial supply and stewardship and therefore have significant challenges gathering truly representative data on resistance prevalence.^14^ These nations also exhibit economic conditions not comparable to SES distributions elsewhere. Because much of their population earns less than $1 USD/day,^15^ socioeconomic measures from LMICs would have limited applicability to those of high-income countries.

## Research question

This review investigates the association between SES and AMR rates across high-income countries based on the World Bank classification, testing the hypothesis that SES is negatively correlated with AMR prevalence. Addressing these disparities will inform policies to mitigate AMR and protect global health.

## Methods

A search from 2015 to 2024 explored databases Embase, PubMed, Web of Science, and CINAHL. Relevant keyword synonyms were compiled. For words such as “antimicrobial” or “resistance,” antimicrobials and drug-resistant bacterial strains were also included specifically, such as “penicillin” or “MRSA.” Complete search terms are in Appendix 1.

Yau et al^12^ conducted a review of AMR between rural and urban communities. Because of its similarity to this research question, the key terms informed synonyms commonly used in titles and abstracts of studies on this topic. The Boolean search structure was organized into 4 components: antibiotic, resistance, incidence/prevalence, and income/SES. Search terms were separated by these 4 sets of synonyms and divided by an AND Boolean, mandating that at least 1 word from each set was present. This ensured a broad range of results while preventing an unmanageable surplus of irrelevant articles. Studies evaluated SES by parameters related to income, occupation, and education. The selection of high-income nations was defined by countries listed as “high-income” according to the World Bank classifications (Table 1). Prevalence of infection or colonization by any AMR bacterial strain was included for all ages of the human population, excluding agricultural and industrial AMR studies. Additional exclusion criteria include non-bacterial infections and populations with significant comorbidities (Table 1). Following extraction, quality assessment was conducted with the Mixed Methods Appraisal Tool Version 2018.^16^

**Table 1.** Inclusion and exclusion criteria

Inclusion criteria	Rationale
Published after January 1, 2015	AMR is quickly evolving, and thus, including only articles published within the last 10 years will ensure that the evidence is up-to-date and reflects the current state of research.
Peer-reviewed primary quantitative research	This ensures utilization of original data rather than secondary analysis or reviews.
Quantitative studies	Quantitative data provides objective and measurable data for identification of trends and statistical analysis.
Study population in high- income countries	High-income country categorization based on World Bank classification.
Human population	This focuses on direct human-associated AMR transmission pathways, reducing confounding from vector-related disease.
Comparison of lower SES group to higher SES group(s)	This enables assessment of socioeconomic disparities in AMR prevalence.
Must measure AMR prevalence and/or incidence rates as a primary outcome	This ensures relevance to the research question by directly addressing the relationship between SES and AMR burden.
Articles written in English were included	This constraint ensures accessibility and accurate interpretation of findings.
Full text available	This allows for comprehensive appraisal of study findings.
Exclusion criteria	Rationale
Investigated fungal, viral, or parasitic drug resistance	The review focuses only on resistant bacteria.
Population includes significant comorbidities or other confounders (eg, HIV, CF, lung transplantation)	Best practice to minimize confounding and other biases.

Note. AMR, antimicrobial resistance; SES, socioeconomic status; HIV, human immunodeficiency virus; CF, cystic fibrosis.

## Results

The database search conducted on May 28, 2024, yielded 1,520 articles, of which 336 duplicates were removed. This left 1,184 articles that underwent the first phase of screening, whereby 26 articles were included and 1,104 were excluded by all 4 reviewers—from May 28, 2024, to June 25, 2024—with 54 conflicts left for resolution. After review, 2 of the 54 articles in contention were included.

**Figure 1.**
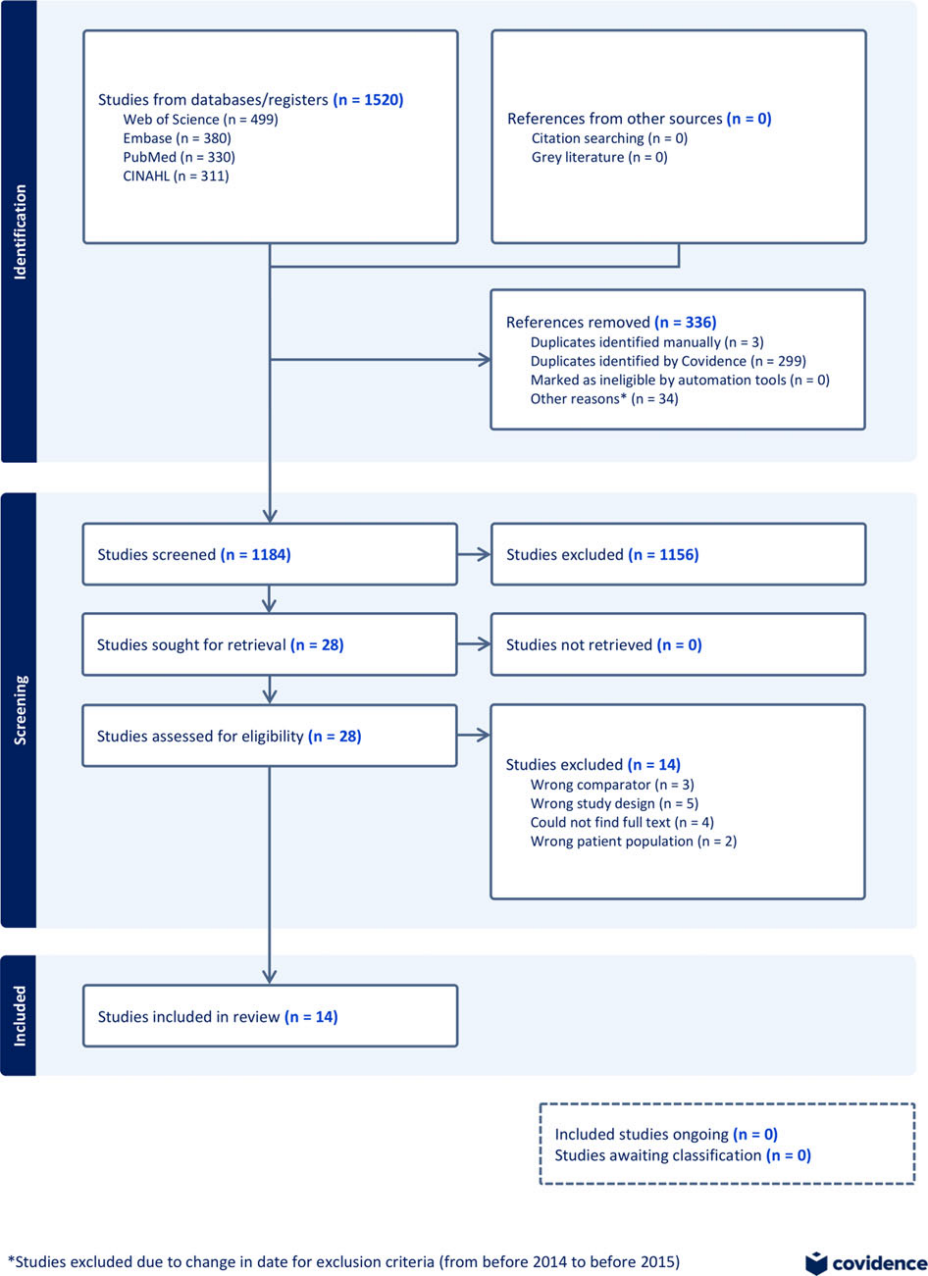
PRISMA diagram of screening process.

In total, 1,156 records were excluded during screening of abstracts and titles, leaving 28 articles for full-text screening (Figure 1). During full-text screening from June 25 to July 13, 2024, 14 studies were included and 14 were excluded by all reviewers without conflicts. Of the 14 included studies,^6,17–29^ 5 were cross-sectional,^6,17–20^ 5 were retrospective cohort,^21–25^ 3 were case-control,^26–28^ and 1 was ecological in study design.^29^ Eight high-income nations were represented: Australia (n = 5),^6,19,21,24,25^ the United States (n = 3),^17,18,26^ France (n = 1),^20^ Canada (n = 1),^22^ Netherlands (n = 1),^23^ Israel (n = 1),^27^ the United Kingdom (n = 1),^28^ and New Zealand (n = 1).^29^

The studies examined a range of infections and resistant organisms (Table 2). The most commonly studied infections were urinary tract infections (UTIs) caused by extended-spectrum β-lactamase (ESBL)-producing or multidrug-resistant (MDR) *E. coli* (n = 6),^19–21,23,26,28^ and MRSA (n = 5).^17,18,22,24,29^ Other pathogens included carbapenem-resistant *Acinetobacter baumannii* (CRAB) (n = 1)^27^ and antibiotic-resistant *H. pylori* (n = 1).^25^ Most studies focused on community-associated infections, though some used hospital-based data or both.

**Table 2.** Study characteristics. Study characteristics and demographics of the included studies (n=14)

Study ID	Study period	Study design	Prevalence/ incidence	Country	Population, sample size	AMR strain	AMR measurement tool	SES measurement tool	Measures of SES
Andreatos^17^	2013– 2015	Cross-sectional	Incidence	United States	26,928 MRSA infections, 3,652 hospitals, 2,982 countries	MRSA bloodstream infection	Medicare Hospital Compare Database	United States Census Bureau	Education, income, room number per house
Blakiston^29^	2013	Ecological	Incidence	New Zealand	18 distinct populations in New Zealand	MRSA and ESBL-*E. coli* infection	Institute of Environmental Science and Research National Survey	New Zealand Index of Deprivation and Ministry of Health publication: Census 2013	Socioeconomic deprivation, household crowding
Casey^26^	2015– 2017	Case-control	Prevalence	United States	325,286 *E. coli* UTI cultures (10.4% MDR)	Multidrug-resistant (MDR) UTI (*E. coli*)	Electronic health record data in Northern California	2011–2015 American Community Survey	Medicaid use, census tract-level socioeconomic deprivation
Chen^18^	2001– 2004	Cross-sectional	Prevalence	United States	18,608 individuals and 207 MRSA cases	MRSA	National Health and Nutrition Examination Survey	National Health and Nutrition Examination Survey	Household income, number in household, insurance
Chua and Stewardson^19^	2017	Cross-sectional	Prevalence	Australia	6,732 *E. coli* isolates	Ceftriaxone- resistant *E. coli*	VITEK MS platform (bioMerieux)	Australian Government Census	IRSD Score 1st decile
Fasugba^21^	2009– 2013	Cohort (retrospective)	Incidence	Australia	146,915 urine samples from 57,837 ACT residents	*E. coli*	ACT pathology laboratory-based data	2006 Australian census data	Residential postcode SEIFA score
Gill^22^	2004– 2014	Cohort (retrospective)	Prevalence	Canada	4,521 community-associated MRSA cases	MRSA	Calgary Laboratory Services records	Statistics Canada’s 2011 Canadian Household Survey	Median household income, employment, education
Henig^27^	2007– 2012	Case-control	Prevalence	Israel	1,190 hospitalized CRAB cases, 1,190 controlled hospitalized patients	CRAB	Clalit Health Services Database	Census socioeconomic deprivation score	Socioeconomic status (low/ intermediate/ high)
Honsbeek^23^	2016– 2017	Cohort (retro spective)	Prevalence	Netherlands	7,966	*E. coli,* *Klebsiella pneumoniae, Proteus mirabilis*	Star-SHL Medical Lab Data	Social and Cultural Planning Office’s SES area score	Average income, education, unemployment
McCowan^28^	2009– 2012	Case-control	Incidence	United Kingdom	1,093,227	*E. coli*	Electronic Communi-cation of Surveillance in Scotland data	Scottish Index of Multiple Deprivation Quintiles	Income, employment, education, health, service access, crime, and housing
Miyakis^24^	2007– 2017	Cohort (retrospective)	Prevalence	Australia	5,929	MRSA	Illawarra Shoalhaven Local Health District Centre for Health Research ICD-10-AM databank	Australian Bureau of Statistics Socio-Economic Indexes for Areas 2011 data set	Index of Relative Socioeconomic Disadvantage score
Paumier^20^	2019	Cross-sectional	Prevalence	France	444,281 *E. coli* isolates	ESBL-*E. coli*	Surveillance and Prevention of Antimicrobial Resistance in Primary Care and Nursing Homes	French National Institute for Statistics and Economic Research	Deprivation index and household crowding
Schubert^25^	1998– 2017	Cohort (retrospective)	Prevalence	Australia	20,108 subjects across 136 of 185 Greater Adelaide Planning Region 1,901 *H. pylori* positive, 797 with antibiotic resistance	*H. pylori*	Test strips using European Committee on Antimicrobial Susceptibility Testing guidelines	2016 National Census Data	SEIFA
Wozniak^6^	2007– 2019	Cross-sectional	Prevalence	Australia	43,448 primary healthcare isolates	*E. coli,* *K. pneumoniae,* *Pseudomonas aeruginosa* and *S. aureus* isolates and their corresponding antibiotic susceptibilities	Primary healthcare clinics; Clinical and Laboratory Standards Institute	Australian Bureau of Statistics	Remoteness, relative socioeconomic disadvantage, average person per household

Note. AMR, antimicrobial resistance; SES, socioeconomic status; MRSA, methicillin-resistant *Staphylococcus aureus*; ESBL-*E. coli*, extended-spectrum β-lactamase–producing *Escherichia coli*; IRSD, Index of Relative Socioeconomic Disadvantage; VITEK MS, automated mass spectrometer system; ACT, Australian Capital Territory; SEIFA, Socio-Economic Indexes for Areas; CRAB, carbapenem-resistant *Acinetobacter baumannii*.

All included studies explored associations between SES and AMR-related outcomes using various proxy measures for SES, including but not limited to income, education, employment, remoteness, insurance status, and census deprivation indices. The indices, when utilized, were often country-specific and locally tailored (Table 3). Regardless, many of these shared common parameters as displayed in Table 4. Among the 14 studies, 12 found at least 1 significant positive association between lower SES and increased AMR risk (Table 3). For example, Casey et al^26^ found that Medicaid usage and neighborhood deprivation were linked to MDR *E. coli* UTIs in a large Californian cohort. Similarly, Henig et al^27^ reported that lower SES was significantly associated with increased risk of CRAB colonization and infection in Israel. Schubert et al^25^ examined antibiotic resistance patterns to *H. pylori* in the Greater Adelaide Region of Australia. 20,108 gastric biopsies identified a 9.45% positivity rate for *H. pylori*, with resistance to metronidazole, clarithromycin, or amoxicillin observed in 41.9% of infections. Multivariate linear regression revealed migration status as a key predictor of *H. pylori* positivity and antibiotic resistance. However, geographically weighted regression found that many independent local regression variables, such as the census socioeconomic index, were not significant for *H. pylori* positivity or associated AMR.^25^

**Table 3.** Summary of findings. Summary of included studies and relevant findings. Measure of interest includes both that of SES and secondary outcomes (AMS). Level of evidence was colored according to significance for ease of interpretation (green, statistically significant; red, not statistically significant). A *P* value < .05 was considered significant

Study ID	Measure of interest	Statistical model	Coefficient value	Level of evidence	Main findings
Andreatos^17^	Antibiotic prescriptions	Multivariate regression coefficient (b)	.432	P < .001	Antibiotic prescription rates are positively associated with MRSA incidence. College education, income above poverty line and median rooms per household were negatively associated with MRSA incidence.
	College education		−.037	P = .024	
	Income above poverty line		−.257	P < .001	
	Median rooms per household		−.107	P < .001	
Blakiston^29^	Living in the lowest socioeconomic quintile	Correlation coefficient (b)	MRSA: .76 ESBL-*E. coli*: .17	P < .01	Living in the most socioeconomically deprived quintile, living in crowded housing and community antimicrobial use positively correlated with MRSA incidence. Living in crowded housing and community antimicrobial use is positively correlated with ESBL-*E. coli* incidence.
				P = .5	
	Living in crowded housing		MRSA: .9 ESBL-*E. coli*: .77	P < .01 P < .01	
	Community antimicrobial use		MRSA: .76 ESBL-*E. coli*: .64	P < .01 P < .01	
Casey^26^	Medicaid use	Relative risk and absolute risk analysis	AR: Increase of 8 additional MDR *E. coli* per 1 000 patients	P < .05	Increased risk of MDR UTI associated with Medicaid and socioeconomic deprivation.
	Socioeconomic deprivation		RR: increase risk of 1.04 and 1.14	P < .05	
Chen^18^	Household income (> $45,000)	Multivariate analyses, odds ratio	.42 (adjusted)	P < .01	High household income was negatively correlated with MRSA prevalence.
	More than 4 individuals in household		.90 (unadjusted)	P = .78	
	Insurance holders		2.15 (unadjusted)	P = .06	
Chua and Stewardson^19^	Index of Relative Socioeconomic Disadvantage 1st Decile	Risk ratio	1.53 (unadjusted) .93 (adjusted)	P < .05	For postcode-level predictors, risk of resistance appeared was inversely associated with remoteness. Residence in the first decile of the IRSD (ie, the most disadvantaged postcodes) was associated with an increased risk of ceftriaxone resistance.
Fasugba^21^	Australian Socio-Economic Indexes for Areas (SEIFA) Socioeconomic Index	Odds ratio (multivariate logistic regression)	1.0	P < .05	There was no positive association between antimicrobial resistance and SES. There were, however, significantly higher odds of resistance in females and older individuals.
Gill^22^	Median household income (> $100,000 CAD)	Risk ratio	.27	P < .0001	High median household income was a protective factor for CA-MRSA infections (negatively correlated).
	Employment rate		.45	P = .1196	
	High education level		1.00	P = .994	
Henig^27^	Socioeconomic status (SES)	Odds ratio	1.87	P = .009	Low SES was associated with an increased risk of carbapenem-resistant *Acinetobacter baumannii* (CRAB) colonization.
			2.18	P = .05	Low SES was associated with an increased risk of CRAB bacteremia.
			1.05	P = .38	Low SES was not significantly associated with an increased mortality among patients with CRAB infection.
Honsbeek^23^	Neighborhood SES Area Score	Odds ratio	4.93	P = .03	Low SES area score was associated with higher resistance to cefuroxime among *E. coli* positive urine samples but was not found to be significant after adjustment for multiple testing.
McCowan^28^	Scottish Index of Multiple Deprivation Quintile	Odds ratio	.83	P < .001	SIMD quintile of the most deprived category (1) had a greater association with blood culture resistance to sentinel antimicrobials compared to those of the least deprived category (5)
Miyakis^24^	Index of Relative Socioeconomic Disadvantage	Multivariate regression analyses	Significant in 39/47 best models	P < .05	Low IRSD score was a key predictor of MRSA infection among admitted patients
Paumier^20^	Deprivation Index (higher score, less deprived)	Adjusted *β1* coefficient	−.115	P < .001	Deprivation was positively correlated with community-acquired ESBL-*E. coli* infections. Overcrowded households were positively correlated with community-acquired ESBL-*E coli* infections.
	Overcrowded households		.049	P < .001	
Schubert^25^	SEIFA Socioeconomic Index	Multivariate regression coefficient (*β*)	.57	P = .353	Increases in SEIFA Socioeconomic Index were not significantly correlated with *H. pylori* positivity or any of its local coefficients for antibiotic resistance.
Wozniak^6^	Remoteness index	Odds ratio (multivariate logistic regression)	5.05 (3GCR-*E. coli*) 5.72 (MRSA)	P < .05	Prevalence of 3rd generation cephalosporin-resistant *E. coli* and MRSA increased with remoteness. Odds of these AMR pathogens increased with higher SES and lower number of persons per household.
	Index of Relative Socioeconomic Disadvantage (OR with higher index value indicate lower disadvantage)		1.93 (3GCR-*E. coli*) 1.35 (MRSA)		
	Average person per household		.40 (3GCR-*E. coli*) .39 (MRSA)		

Note. MRSA, methicillin-resistant *Staphylococcus aureus*; ESBL-*E. coli*, extended-spectrum β-lactamase–producing *Escherichia coli*; MDR, multidrug resistant; UTI, urinary tract infection; AR, absolute risk; RR, relative risk; IRSD, Index of Relative Socioeconomic Disadvantage; SEIFA, Socio-Economic Indexes for Areas; SES, socioeconomic status; AMR, antimicrobial resistance.

**Table 4.** Determinants of SES and correlation to AMR. Compiled list of SES measures extracted from included studies. N refers to the number of studies measuring the given SES factor

SES measure	N	Number of significant correlations*	Relationship to AMR
Income	3	3	High household income was a protective factor for all 3 studies involved. Conversely, low household income was positively correlated with AMR incidence/prevalence.
Household crowding	5	3	Household crowding was positively associated in 3 of5 studies. One study found no significant correlation^18^ and another found an inverse correlation with persons per household.^6^
Deprivation Index or Socioeconomic Status Index	11	9	Relative socioeconomic deprivation/disadvantage was positively correlated to AMR prevalence in 9 of 11 studies. Blakiston et al^29^ was among the 9 reviewed as a positive correlation, although only 1 of the 2 tested resistant strains yielded a statistically significant result. Fasugba et al^21^ found that high-SES was associated with the same odds of multidrug-resistant UTI as those in low-SES populations. Wozniak et al^6^ stated that their finding of high-SES association with increased prevalence of AMR had significant limitations.
Education	2	1	College education was a protective factor for AMR in one study.^17^ Low education was positively correlated to AMR, until adjusted for antimicrobial use, in which education failed to retain its association with AMR. There was no association between higher education status and AMR infections in another study.^22^
Antibiotic use/prescription rates	2	2	Rates of antibiotic prescriptions and use in the community was positively associated with AMR.^17,29^
Medicaid dependence	1	1	Medicaid use was associated with a small but significant increase in AMR prevalence.^26^
Private insurance	1	0	Private insurance holders had lower rates of AMR but not to a statistically significant degree.^18^
Employment	1	0	There was no association with AMR.^22^
Remoteness	1	1	There was a positive correlation between remoteness and AMR.^6^

Note. AMR, antimicrobial resistance; UTI, urinary tract infection; SES, socioeconomic status.

* This column indicates results in supportive affirmation of the hypothesis, not the number of statistically significant findings indicated by red and green in Table 3. For example, a negative correlation between income and AMR prevalence would be a supportive finding, whereas a positive correlation between household crowding and AMR would be affirmative.

Several studies included additional SES-related variables such as household crowding, remoteness, and proportion of residents born outside the country, which were positively associated with AMR.^6,19,21,25–29^ Furthermore, Blakiston et al^29^ and Chua and Stewardson^19^ identified both community antimicrobial use and household density as significant contributors to MRSA and ESBL-*E. coli* incidence. Age and sex were also explored, with older women found to have an increased risk of MDR UTIs in an Australian cohort.^21^

Most studies were observational in nature and geographically concentrated in Australia^6,19,21,24,25^ and North America.^17,18,22,26^ Ecological and cross-sectional designs were common, limiting causal inference. Nevertheless, consistent associations across multiple regions and methodologies suggest a robust relationship between SES and AMR prevalence.

## Determinants of socioeconomic status

Included studies investigated a plethora of SES determinants: income, household crowding, education, Medicaid use, insurance, employment, and census-derived deprivation scores (Table 4). Although these determinants are collectively a comprehensive measure of SES, direct comparison between studies is made difficult by their heterogeneity. Overall, 12 of 14 studies reported at least 1 significant association between a determinant of SES deprivation and increased AMR prevalence (Table 3). Of the 2 studiesthat did not find any significant positive correlations,^6,25^ Wozniak et al^6^ found that AMR prevalence increased with remoteness, independent of socioeconomic indices or income. Although remoteness may relate to SES, it was not considered as an independent determinant. Schubert et al^25^ reported no significant increase in *H. pylori* positivity or any local coefficients for antibiotic resistance with increasing SEIFA Socioeconomic Index. Across the 14 included studies, 9 unique measures including and related to SES were investigated in 27 iterations (N, Table 4). Overall, 20 instances of the 27 were statistically significant correlations in support of the hypothesis (Table 4). Hence, most studies report positive correlations between SES and AMR in a majority of the various SES determinants, but further analysis is required to ascertain which determinants are the largest contributors and develop understanding of a potential dose-response relationship.

## Mechanisms of association between SES determinants and AMR

Household crowding was a key determinant of SES with a demonstrated causal mechanism of low SES and increased AMR prevalence.^17,20,26,29^ Blakiston et al^29^ identified increased prevalence of ESBL-*E. Coli* and MRSA with crowded housing, organisms that are known to readily spread within households. However, Wozniak et al^6^ challenged simplistic crowding metrics of “persons per household,” emphasizing the need to assess housing quality rather than simply the number of occupants. Community antibiotic use also played a role, with Andreatos et al^17^ and Blakiston et al^29^ reporting higher prescription rates in lower SES areas, driving MRSA and ESBL-*E. coli* resistance. In contrast, Wozniak et al^6^ rejected antibiotic use as a sole driver, citing increased AMR prevalence despite longstanding stewardship programs in remote Australia.

Higher income appeared protective, with Chen et al^18^ and Gill et al^22^ showing inverse relationships between affluence and MRSA infections. Similarly, McCowan et al^28^ found more antimicrobial-resistant *E. coli* in Scotland’s most deprived areas but did not propose specific mechanisms. Henig et al^27^ and Wozniak et al,^6^ however, framed AMR disparities as a function of healthcare access rather than income alone. Remoteness was a more complex variable. Univariable analysis for remoteness showed a significant association with AMR for Chua and Stewardson,^19^ but findings were not significant in multivariate regression. Wozniak et al^6^ argued that remoteness itself directly contributed to AMR spread in resource-limited settings, underscoring the need for context-specific, targeted approaches to AMR mitigation rather than one-size-fits-all interventions.

Overall, these studies suggest that no single mechanism fully explains SES-related AMR disparities. Instead, determinants such as household density, antibiotic exposure, income, and healthcare accessibility interact in complex ways, necessitating tailored interventions that consider both local socioeconomic determinants and healthcare policies.

## Discussion

This systematic review provides evidence for a more complex correlation between low SES and AMR prevalence in high-income nations. Across the included studies, multiple SES determinants such as income, household crowding, and community antibiotic use were associated with increased AMR burden. Community antibiotic prescribing patterns were also linked to AMR prevalence, particularly in lower-income communities, though causality remains uncertain.

This review also highlights the need for targeted public health interventions that address a multitude of factors, from social and behavioral determinants that could increase risk of infection to access to safe and appropriate healthcare at a population level. This goes beyond the scope of traditional AMS programs, which have primarily focused on reducing the quantum of inappropriate prescribing in hospital settings.^8,30^ Remoteness and rurality as a driver of AMR have also been the primary focus of targeted AMS programs in the past.^30^ Such programs, however, could potentially overlook socioeconomically disadvantaged urban communities and the significant burden of resistance they pose. Examples could include investing in affordable housing construction, maintenance, and subsidizations for ventilation and sanitation hardware.^31^ Public education on hygiene and antibiotic use from junior to senior schools also shows promise as evidenced by the e-Bug program in Europe which significantly improved knowledge and awareness in young people.^32^

## Strengths and limitations

We observed a heterogeneity of study types and settings among included studies, from hospitalized patients to community screening participants. The included studies were well-conducted, with large sample sizes and broad geographic coverage, enhancing the external validity of the findings (Table 5). However, several limitations are present. No formal publication bias assessment was conducted, and therefore, publication bias remains a potential limitation of this review. One study used an ecological design, which is vulnerable to ecological fallacy. Although this does not invalidate the findings, it constrains their applicability to policies targeting AMR at the individual level.

**Table 5.** Quality assessment of included studies. The Mixed Methods Appraisal Tool Version 2018^16^ was used to evaluate the quality of each study

Author	3.1	3.2	3.3	3.4	3.5	Overall
Andreatos^17^	Yes	Yes	Yes	Yes	**No**	Moderate
Blakiston^29^	Yes	Yes	**No**	Yes	Yes	Moderate
Casey^26^	Yes	Yes	**No**	Yes	Yes	Moderate
Chen^18^	Yes	Yes	Yes	Yes	Yes	High
Chua and Stewardson^19^	Yes	Yes	Yes	Yes	Yes	High
Fasugba^21^	Yes	Yes	Yes	Yes	Yes	High
Gill^22^	Yes	Yes	Yes	**No**	Yes	Moderate
Henig^27^	Yes	Yes	Yes	Yes	Yes	High
Honsbeek^23^	Yes	Yes	Yes	Yes	Yes	High
McCowan^28^	Yes	Yes	Yes	Yes	Yes	High
Miyakis^24^	Yes	Yes	Yes	Yes	Yes	High
Paumier^20^	Yes	Yes	Yes	Yes	Yes	High
Schubert^25^	Yes	Yes	Yes	Yes	Yes	High
Wozniak^6^	Yes	Yes	Yes	Yes	Yes	High

The wide variation in measurement of variables among studies included is the primary limitation in this review. The definition of AMR as the outcome variable varied considerably, not only in the studied strain but also in the outcome classification. Some studies defined AMR through the presence of a resistant microbial in a community screening test, while other studies were based on positive diagnostic findings of hospitalized cases of UTI or bacteremia. A standardization of SES measurements within a nation, from individual to population levels, would also facilitate research and benchmarking across health boards and between different states. We recognize that this would allow robust meta-analyses, which could not be conducted due to the heterogeneity of SES and AMR measures in the current literature.

## Conclusion

This systematic review identifies socioeconomic determinants as a potentially significant driver of AMR. Investigating the spread of resistance in low-SES populations of both urban and rural communities presents a new paradigm for AMS programs, which have previously focused primarily on inappropriate prescription and remote communities.^30^ We recommend public health initiatives prioritize infection control and AMS in populations of lower SES, both urban and rural, where risks of AMR may be heightened.

AMR is a multifaceted issue influenced by SES disparities, healthcare accessibility, and antibiotic exposure. Addressing these disparities will require collaborative, multi-sectoral approaches that integrate public health, healthcare policy, and socioeconomic interventions. Future AMR research will benefit from adopting a standardized demographic index for SES to enable more cross-study comparisons and meta-analysis for policy relevance. Concurrently, prospective studies that clarify causal pathways should be conducted to tailor more effective strategies to mitigate AMR in socioeconomically disadvantaged communities. These recommendations, when implemented, would ultimately promote health equity, prevent AMR spread, and strengthen global efforts to reduce AMR.

## Supporting information

10.1017/ash.2025.10177.sm001 Levitch et al. supplementary material Levitch et al. supplementary material

## Research transparency and reproducibility

The data described in this article are all publicly available.

## Supplementary material

The supplementary material for this article can be found at https://doi.org/10.1017/ash.2025.10177.
