# Supplementary material for: The impact of socioeconomic status on the prevalence of antimicrobial resistance in high-income nations: a systematic review
Source: Antimicrob Steward Healthc Epidemiol. 2025 Oct 14;5(1):e264. doi: 10.1017/ash.2025.10177 (PMC12538351; doi:10.1017/ash.2025.10177)
Supplement: Levitch et al. supplementary material [file S2732494X25101770sup001.docx]

**Appendix 1. Complete Database Search Terms**

| Database | Advanced Search Terms |
| --- | --- |
| CINAHL | AB (antimicrobial* OR antibiotic* OR macrolide* OR “beta lactam” OR penicillin* OR cephalosporin* OR azithromycin* ) AND AB (resistan* OR “Antimicrobial resistance” OR “AMR” OR “MDRO” OR “multidrug-resistant organisms” OR “MRSA” OR “methicillin-resistant staphylococcus aureus” OR “VRE” OR “vancomycin-resistant enterococcus” OR “azithromycin resistan*” OR “Antibiotic resistan*” OR “drug resistan*” OR “resistant bacteria”) AND AB ( use OR consumption OR stewardship OR prescri* OR dispens* OR“prevalence” OR “incidence” ) AND AB ( "socioeconomic" OR "income" OR "SES" OR "poverty" OR “socio-economic”) |
| Embase | ('antibiotic resistance'/exp OR 'antimicrobial resistance' OR 'macrolide'/exp OR 'beta lactam'/exp OR 'penicillin'/exp OR 'cephalosporin'/exp OR 'azithromycin'/exp OR ‘macrolide’/exp) AND (‘drug resistance’/exp OR 'multidrug resistance'/exp OR 'mrsa':ab,ti OR 'methicillin resistant staphylococcus aureus':ab,ti OR 'vancomycin resistant enterococcus':ab,ti OR 'carbapenem-resistant enterobacteriaceae':ab,ti) AND ('bacterial infection'/exp OR 'bacterial pneumonia'/exp) AND ('prevalence':ab,ti OR 'consum*':ab,ti OR 'dispens*':ab,ti OR 'distribut*':ab,ti OR 'prescri*':ab,ti OR 'moralit*':ab,ti OR 'sepsis'/exp OR 'septic shock'/exp OR 'case fatality rate'/exp) AND ('socioeconomic status':ab,ti OR 'socio-economic status':ab,ti OR 'socioeconomic':ab,ti OR ‘socio-economic’:ab,ti OR 'income':ab,ti) |
| Pubmed | "drug resistance, microbial"[MeSH Terms] OR "macrolides"[MeSH Terms] OR "beta Lactams "[MeSH Terms] OR "penicillin" [MeSH Terms] OR  "Cephalosporins "[MeSH Terms] OR "azithromycin" [MeSH Terms] OR  "drug resistance, multiple” [MeSH Terms] OR "MRSA" [Title/Abstract] OR "methicillin resistant staphylococcus aureus" [MeSH Terms] OR "vancomycin resistance" [MeSH Terms] OR “VRE” [MeSH Terms]) **AND** ("staphylococcal skin infections"[MeSH Terms] OR "soft tissue infections"[MeSH Terms] OR "bacterial infections"[MeSH Terms] OR "bacterial pneumonia"[Title/Abstract] OR "urinary tract infections"[MeSH Terms]) **AND** ("prevalence"[Title/Abstract] OR "consum*"[Title/Abstract] OR "dispens*"[Title/Abstract] OR "distribut*"[Title/Abstract] OR "prescri*"[Title/Abstract] OR "mortali*"[Title/Abstract] OR "sepsis"[Title/Abstract] OR "septic"[Title/Abstract] OR "case fatality rate"[Title/Abstract]) **AND** ("social class"[MeSH Terms] OR "socioeconomic factors"[MeSH Terms] OR "socioeconomic"[Title/Abstract] OR  "income"[Title/Abstract]) |
| Web of Science | AB=(antimicrobial* OR antibiotic* OR macrolide* OR  "beta lactam" OR penicillin* OR ce*alosporin OR azithromycin*)  AND AB=(resistan* OR "MDR" OR "multi-drug resistan*" OR "MRSA" OR  "methicillin-resistant staph* aureus" OR "VRE" OR "vancomycin-  resistant enterococc*" OR "CRE" OR "carbapenem-resistant  enterobacteria*" OR "AMR" OR "antimicrobial resistan*")  AND AB=(“prevalence” OR “incidence”)  AND AB=("SES" OR "socioeconomic status" OR "socio-  economic status" OR "socioeconomic" OR "income") |

**Figure 1. PRISMA Diagram of Screening Process**

(Attached separate file)
